# Urine NGAL and KIM-1: tubular injury markers in acute lymphoblastic leukemia survivors

**DOI:** 10.1007/s00280-020-04164-3

**Published:** 2020-10-14

**Authors:** Eryk Latoch, Katarzyna Konończuk, Katarzyna Taranta-Janusz, Katarzyna Muszyńska-Rosłan, Edyta Szymczak, Anna Wasilewska, Maryna Krawczuk-Rybak

**Affiliations:** 1 Department of Pediatric Oncology and Hematology, Medical University of Bialystok, ul. Waszyngtona 17, 15-274 Białystok, Poland; 2 Department of Pediatrics and Nephrology, Medical University of Bialystok, Białystok, Poland

**Keywords:** Urine KIM-1, Urine NGAL, Acute lymphoblastic leukemia, Chronic kidney diseases, Childhood cancer survivors, CCS

## Abstract

**Introduction:**

Nephrotoxicity is a potential adverse effect of anticancer treatment in childhood. Cytostatics, abdominal radiotherapy, total body irradiation (TBI) and some agents used in supportive care may induce acute kidney injury (AKI) or lead to chronic kidney disease (CKD). The aim of this study was to test the hypothesis whether urinary kidney injury molecule-1 (KIM-1) and neutrophil gelatinase-associated lipocalin (NGAL) are increased in acute lymphoblastic leukemia (ALL) survivors.

**Method:**

The study cohort consisted of 86 patients (42 females) previously treated for ALL. The median time after cessation of treatment was 6.55 (IQR: 1.96–9.93) years and median age at the time of study: 12 (IQR: 6.76–16.00). The control group included 53 healthy peers. Immunoenzymatic ELISA commercial kits were used to measure urine KIM-1 and NGAL levels.

**Results:**

The median levels of urine uNGAL (p < 0.05), uNGAL/creatinine (cr.) ratio (*p* < 0.0001) and uKIM-1/creatinine ratio (*p* < 0.0001) were significantly higher in ALL survivors in comparison with healthy controls. Female patients had significantly higher levels of NGAL and NGAL/cr. than males (mean 8.42 ± 7.1 vs. 4.59 ± 4.5 ng/mL and 86.57 ± 77 vs. 37.7 ± 37 ng/mg, respectively; p < 0.01). Of all the study participants, 11 (13%) presented eGFR below 90 mL/min/1.73 m^2^. The NGAL/cr. ratio seemed to be the best predictor of decreased eGFR (AUC = 0.65). The cumulative dose of methotrexate and cyclophosphamide did not predict the values of the urine NGAL, NGAL/cr., KIM-1/cr. and eGFR.

Five years after the end of treatment, the patients had higher levels of uKIM-1 (1.02 ± 0.8 vs. 0.62 ± 0.6 ng/mL, *p* < 0.01), uNGAL (7.9 ± 6.7 vs. 4.6 ± 5 ng/mL, *p* < 0.01) and lower eGFR (114 ± 29 vs. 134 ± 35 mL/min/1.73 m^2^, *p* < 0.01) in comparison with ALL survivors with the observation period of less than 5 years.

**Conclusion:**

We demonstrated that ALL survivors have higher levels of urine NGAL**,** NGAL/cr. and uKIM-1/cr. ratio as compared to the control group. Further long-term follow-up studies are necessary to assess the significance of the NGAL and KIM-1 and their relationship to kidney damage after anticancer treatment in childhood.

## Introduction

In the last years, progress in the diagnosis and treatment of childhood cancer has resulted in up to 80% of patients achieving remission or permanent healing. Through long-term observations of patient cohorts, we have learned a lot about the early and long-term effects of anticancer treatment. Nowadays, the main goal, apart from achieving the best possible treatment outcome, is to maintain good health after therapy termination. Nephrotoxicity (glomerulopathy, tubulopathy) is a potential late effect of cancer treatment in childhood. It is well known that some cytostatic agents such as cisplatin, ifosfamide and abdominal radiotherapy or total body irradiation (TBI) may induce acute kidney injury (AKI) and/or long-term deterioration of kidney function [1–5].

Acute lymphoblastic leukemia (ALL) is the most common neoplastic disease in childhood and shows an overall survival between 80 and 98% depending on the risk group [6]. The most nephrotoxic cytostatics (cisplatin, ifosfamide) are not included in leukemia treatment protocols. However, other anticancer agents such as methotrexate or cyclophosphamide that are widely used may impair kidney function. Additionally, some antibiotics (e.g., aminoglycosides, vancomycin, amphotericin), proton pump inhibitors, analgesics (e.g., paracetamol, ibuprofen) as well as contrast media used in computed tomography may induce kidney injury. In our previous study assessing the prevalence of late effects of anticancer treatment in Poland, 88.25% of survivors had one or more symptoms/complaints indicating dysfunction of at least one organ. Of all patients treated for ALL in that study, 24.6% presented with symptoms suggestive for urinary tract dysfunction (information was collected on the basis of: medical history, questionnaire, physical examination and laboratory results). No severe or life-threatening health conditions (3rd and 4th grades according to CTCAE) were observed, yet there were subclinical or first-degree symptoms [7].

Serum creatinine level and estimated glomerular filtration rate (eGFR) are the simplest and most commonly used methods for assessing kidney function. On the other hand, several limitations of this method exist due to the influence of, among others, muscle mass, age, sex and hydration status. Moreover, the deterioration of kidney function may be very long unnoticed in clinical and laboratory tests, as it is often the case in chronic kidney disease (CKD). For this reason, new biomarkers are constantly being sought to reflect in particular the initial stage of kidney damage. In recent years, new biomarkers have been used to detect early onset of acute kidney injury, such as neutrophil gelatinase-associated lipocalin (NGAL), a member of the lipocalin family, and kidney injury molecule-1 (KIM-1), type 1 transmembrane glycoprotein.

NGAL is detected in various types of human tissues including kidneys (monomeric form), gastrointestinal and respiratory tract. Furthermore, its neutrophil dimeric form also participates in the immunological response. In healthy subjects, it is present in serum and urine at very low levels. Due to the low molecular weight and low degradation, NGAL is excreted into the urine (uNGAL) and its concentration increases immediately in the first hours after AKI. In kidneys, NGAL is excreted from the loop of Henle epithelial cells, the proximal and distal tube, and its level increases in line with the degree of renal tubular damage (toxic, ischemic) [8]

Kidney injury molecule-1 (KIM-1) is not detectable in health conditions. However, in the case of acute kidney damage, its excretion in proximal tubule cells in response to AKI is significantly increased [9–13]. Essentially, KIM-1 is useful as a marker of proximal tubular injury, whereas NGAL is a marker of proximal and distal tubule damage in response to various types of injury (i.e., ischemic, toxic, inflammation).

The available reports about the applicability of NGAL and KIM-1 as prognostic factors for early stages of chronic kidney damage are conflicting [12, 14–17]. In the literature, NGAL and KIM-1 levels have been studied as biomarkers detecting kidney injury during anticancer treatment with cytostatics such as ifosfamide and/or cisplatin. However, there are no studies assessing the usefulness of these markers in detecting early stages of CKD in long-term childhood cancer survivors [18–20].

The aim of the present study was to test the hypothesis that urinary kidney injury molecule-1 (KIM-1) and neutrophil gelatinase-associated lipocalin (NGAL) are increased in survivors of childhood acute lymphoblastic leukemia many years after treatment.

## Materials and methods

### Study population

A total of 86 out of 106 patients treated between 1996 and 2007 in childhood for acute lymphoblastic leukemia were enrolled in the study. Eligible patients included any individual who visited the outpatient clinic for a follow-up after treatment completion. All of them were in complete first continuous remission. Exclusion criteria were: relapse, hematopoietic stem cell transplantation, solitary functional kidney, any congenital anomalies of kidney or urinary tract, and current infection. All the children were treated in accordance with the international Berlin–Frankfurt–Münster (BFM) protocols approved by the Polish Pediatric Leukemia and Lymphoma Group. Written informed consent was obtained from the participants or their parents/guardians. The study was approved by Ethical Committee of the Medical University of Bialystok in line with the Declaration of Helsinki (permission number: R-I-002/62/2018).

For all subjects, clinical history including demographic information, coexisting diseases, previous anticancer treatment, cumulative dosage of nephrotoxic drugs (cyclophosphamide, methotrexate) and radiotherapy was obtained from medical records. The total cumulative glucocorticoids dosage was calculated with prednisone equivalent [21]. Every patient underwent full physical examination. Weight was measured on an electronic scale (Seca, Germany) and height was taken using a Martin anthropometer. Body mass index (BMI) was calculated according to the formula: weight [kg]/height^2^ [m^2^]. Blood pressure was measured using a standardized sphygmomanometer (performed three times at 1–2-min intervals); before the measurement, the participant rested peacefully for 5 min. The average values of the second and third measurements of systolic blood pressure (SBP) and diastolic blood pressure (DBP) were used for subsequent analyses. Hypertension (HT) was defined as a mean SBP and/or DBP level ≥ 95th percentile adjusted for age, sex and height [22].

The control group consisted of 53 healthy peers (29 female) with a pair of normal kidneys, who were offspring of the Pediatric and Nephrology Department’s employees and healthy school pupils recruited form the OLAF study [23]. All the study participants had a normal ultrasound examination of the kidney and urinary tract performed by an experienced pediatric radiologist.

### Biochemical analysis

All laboratory tests were performed after 12-h night fasting. The biochemical analyses included an assessment of serum creatinine level (enzymatic method) and the estimated glomerular filtration rate (mL/min/1.73 m^2^) calculated using the updated Schwartz formula: eGFR = 0.413 × (height in cm / serum cr. in mg/dL). Clean catch urine samples were collected in the morning and stored at − 80ºC. Commercial immunoassays (R&D SYSTEMS a bio-techne brand, Quantikine^®^ ELISA, Minneapolis, USA) were used to measure urine KIM-1 and NGAL levels and the values were expressed in nanograms per milliliter. All specimens were diluted to obtain the concentration for the optimal measurement in accordance with the instructions for the ELISA kit. Due to the fact that different urine dilutions may affect the concentration of the tested biomarkers, we calculated both urine KIM-1 and NGAL levels per milligram urine creatinine (cr.) (KIM-1/cr. and NGAL/cr. ratios, respectively). Urine albumin concentration was determined by Lowry’s method and the results were expressed in μg/mL. Children with a urinary albumin/creatinine ratio between 30 and 300 μg/mg were considered to have albuminuria. All the measurements were done after the end of anticancer treatment during follow-up visit (at least 6 months after completion of treatment).

### Statistical analysis

Statistical analysis was performed using STATA 12.1 version (StatCorp, College Station, Texas, USA). Data were expressed as median (Me) and quartiles (Q) or mean and standard deviation (SD) when appropriate. All continuous variables were tested for normal distribution using Shapiro–Wilk or Kolmogorov–Smirnov tests. In the univariate analysis, Fisher exact test and Chi square (*χ*^2^) test were used. Continuous variables were compared with the Wilcoxon rank-sum or *t* Student test depending on the normal distribution. Univariate and multivariate logistic regression models with urine KIM-1/cr. and NGAL/cr. as independent variables were created. The correlations between variables were calculated by Spearman or Pearson correlation coefficients. The diagnostic value of the biomarkers and the optimum cut-off values were determined based on receiver operating characteristic (ROC) analysis, known as the area under the curve (AUC). Based on a rough classifying system, AUC was interpreted as follows: 90–100 (excellent), 80–90 (good), 70–80 (fair), 60–70 (poor), < 60 (fail). A *p* value below < 0.05 was considered significant.

## Results

The clinical characteristics of study and control group are shown in Table 1. The median age at the time of diagnosis was 4.24 years (range: 0.5–17.9 years), while the median time from treatment cessation to follow-up was 6.55 ± 5.1 years (range 0.5–22.8 years). Age and sex did not differ between the study and reference group.

**Table 1:** Clinical characteristics of the survivors of acute lymphoblastic leukemia (ALL) and control group

	ALL		Control group	
	Number (%)^a^	Median (IQR)^c^	Number (%)^a^	Median (IQR)^c^
Patients	86 (100)		53 (100)	
Male	44 (51.1)		24 (45.3)	
Female	42 (48.9)		29 (54.7)	
Age at diagnosis (years)	86 (100)	4.24 (2.86–6.96)		
Age at study (years)	86 (100)	12 (6.76–16.00)	53 (100)	11.5 (7.45–15.24)
Follow-up after treatment (years)	86 (100)	6.55 (1.96–9.93)		
Chemotherapy^e^	86 (100)			
Methotrexate (cumulative dose in mg/m^2^)		8000 (8000–8000) 10,050 ± 5494^b^		
Cyclophosphamide (cumulative dose in mg/m^2^)		3000 (3000–3000) 3620 ± 2349^b^		
Glucocorticoids (cumulative dose in mg/m^2^)^d^		3087 (3087–3087) 3314 ± 944.3^b^		
Radiotherapy				
Cranial radiotherapy (CRT) (cumulative dose in *Gray*)	9 (10.5)	12 (12–12) 12.5 ± 1.73^b^		
No	77 (89.5)			

*ALL* acute lymphoblastic leukemia

*p < *0.05*

^a^Percent of the total

^b^Mean and standard deviation (SD)

^c^Median and interquartile range (IQR)

^d^Calculated as prednisone equivalents

^e^Most patients received the same dosage of anticancer agents according to the treatment protocol; therefore, the first and third quartiles did not differ from the median

Compared to the control group, the ALL patients had higher values of creatinine level (0.57 vs. 0.49, *p* < 0.05), although the difference was not shown in the eGFR value (122 ± 33 vs. 124 ± 37 mL/min/1.73 m^2^, *p* > 0.05). The summary of the biochemical parameters of all studied patients is presented in Table 2. All subjects had normal eGFR prior to treatment and no differences were found compared to the control group.

**Table 2:** Summary of the biochemical parameters of all studied patients

Total	ALL survivors	Control group	*p*
	86	53	
Serum creatinine (mg/dL)	0.57 (0.42–0.74)	0.49 (0.4–0.63)	< 0.05
eGFR (mL/min/1.73 m^2^)	115.8 (100–140.9)	118.1 (102.9–135.6)	0.875
KIM (ng/mL)	0.59 (0.19–1.34)	0.81 (0.41–1.04)	0.335
KIM/cr. (ng/mg cr.)	5.71 (3.06–8.64)	0.92 (0.44–1.48)	< 0.0001
NGAL (ng/mL)	4.85 (1.92–7.96)	0.004 (0.001–0.005)	< 0.05
NGAL/cr. (ng/mg cr.)	42.11 (13.85–76.34)	0.004 (0.001–0.007)	< 0.0001

*ALL* acute lymphoblastic leukemia*, eGFR* estimated glomerular filtration rate, *KIM-1* urinary kidney injury molecule-1, *NGAL* urinary neutrophil gelatinase-associated lipocalin, *cr.* creatinine

Data are given as the median with interquartile range

**Fig. 1:**
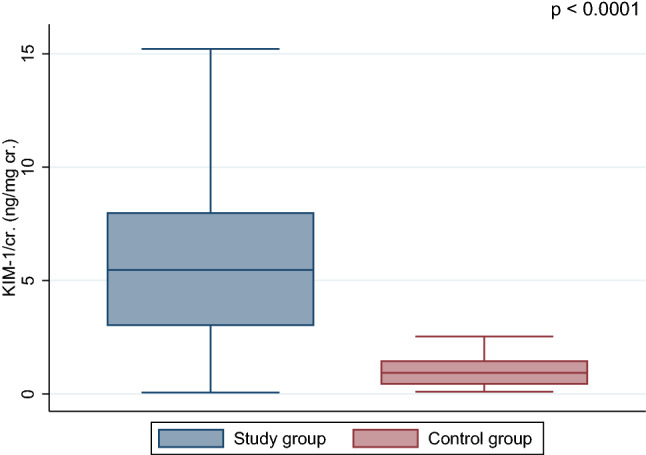
Comparison of the urine KIM-1/cr. (kidney injury molecule-1/creatinine) between acute lymphoblastic leukemia survivors and control group

**Fig. 2:**
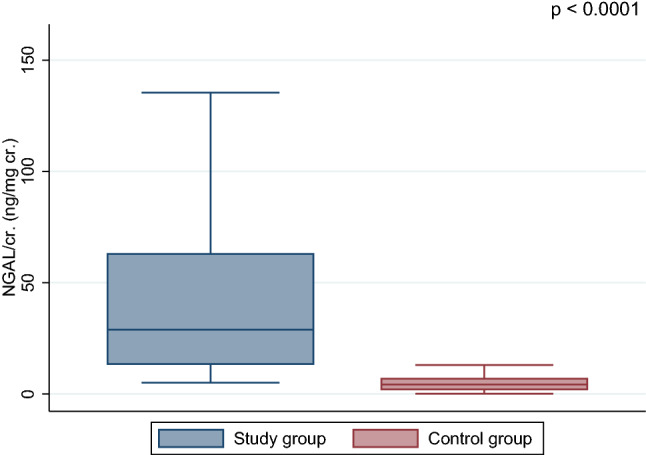
Comparison of the urine NGAL/cr. (neutrophil gelatinase-associated lipocalin/creatinine ratio) between acute lymphoblastic leukemia survivors and control group

**Fig. 3:**
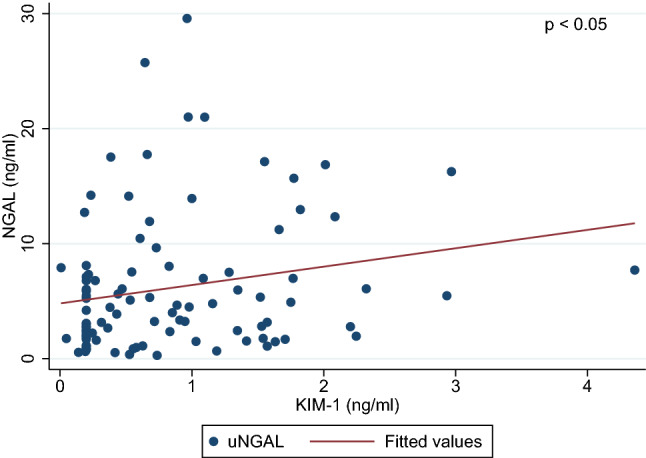
Correlations between the urine neutrophil gelatinase-associated lipocalin (NGAL) and the kidney injury molecule-1 (KIM-1)

Both ratios, KIM-1/cr. and NGAL/cr., were significantly higher in ALL survivors in comparison with healthy controls, *p* < 0.0001 (Figs. 1 and 2). Moreover, NGAL levels (ng/mL) were found to be higher in the study group (mean 6.46 ± 6.2 vs. 0.005 ± 0.004, *p* < 0.0001), but not KIM-1 (mean 6.87 ± 6.1 vs. 0.81 ± 0.4 ng/mL, *p* = 0.335). In CCS, we found a positive correlation between KIM-1 and NGAL concentration (*r* = 0.21, *p* < 0.05) (Fig. 3). Female patients had significantly higher level of NGAL and NGAL/cr. ratio than males (mean 8.42 ± 7.1 vs. 4.59 ± 4.5 and 86.57 ± 77 vs. 37.7 ± 37, respectively; *p* < 0.01) but no differences in concentration of KIM-1 and KIM-1/cr. ratio were found.

Among all the study participants, there were 11 (13%) who presented eGFR below 90 but over 60 mL/min/1.73 m^2^ (Table 3). Higher values of NGAL/cr. ratio were found in the subgroup of patients with decreased eGFR in comparison with those who had normal eGFR (74 ± 34 vs. 61 ± 68 ng/mg, *p* < 0.05). There were no differences in KIM-1 or KIM-1/cr. levels between groups depending on eGFR. Furthermore, no link was found between albuminuria and all the above parameters.

**Table 3:** Urine concentration of biochemical parameters in acute lymphoblastic leukemia (ALL) survivors according to estimated glomerular filtration rate (eGFR)

Total	eGFR > 90 mL/1.73 m^2^	eGFR < 90 mL/1.73 m^2^	*p*
	75 (87%)	11 (13%)	
KIM-1 (ng/mL)	0.63 (0.20–1.40)	0.24 (0.19–1.14)	0.483
KIM-1/cr. (ng/mg cr.)	5.92 (3.00–9.10)	4.76 (2.79–6.78)	0.545
NGAL (ng/mL)	4.43 (1.83–10.25)	6.79 (3.28–7.80)	0.496
NGAL/cr. (ng/mg cr.)	32.04 (13.48–71.88)	69.93 (51.05–98.39)	< 0.05

*eGFR* estimated glomerular filtration rate, *KIM-1* urinary kidney injury molecule-1, *NGAL* urinary neutrophil gelatinase-associated lipocalin, *cr.* creatinine

Data are given as the median with interquartile range

ROC analyses were conducted to assess the diagnostic profile of KIM-1 and NGAL in identifying patients with an impaired renal function (eGFR < 90 mL/min/1.73 m^2^). We found that NGAL/cr. showed poor diagnostic factor profile, describing an area under curve (AUC) of 0.65 (95% CI: 0.49–0.79) with the best cut-off value of 19.14 ng/mg (sensitivity 100%, specificity 32.3%), whereas KIM-1/cr. turned out to be a fail predictor of reduced eGFR (AUC 0.44; 95% CI: 0.25–0.62).

**Fig. 4:**
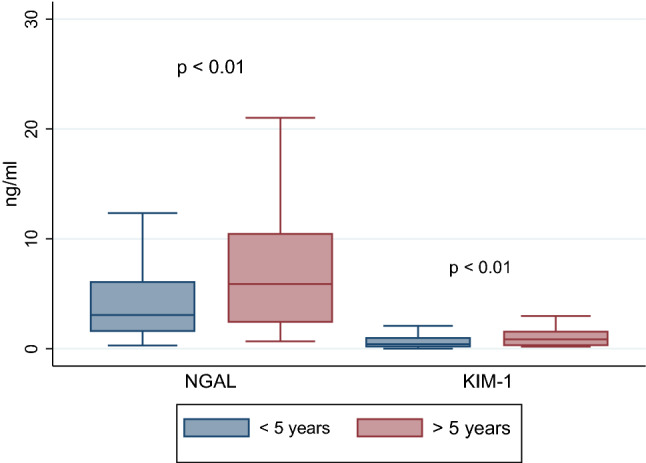
Urine NGAL/cr. (neutrophil gelatinase-associated lipocalin/creatinine ratio) and the KIM-1/cr. (kidney injury molecule-1/creatinine ratio) according to the time after completion of treatment (below and above 5 years)

Given the fact that children in the first years of life might be more vulnerable to drug-induced nephrotoxicity, the study group was divided according to the time of diagnosis (0.5–3 years. vs. 3 years. and above); however, no differences in the concentration of the analyzed biomarkers were found. Interestingly, forty-eight subjects over 5 years after the end of treatment had higher levels KIM-1 (1.02 ± 0.8 vs. 0.62 ± 0.6 ng/mL, *p* < 0.01), NGAL (7.9 ± 6.7 vs. 4.6 ± 5, *p* < 0.01) and lower eGFR (114 ± 29 vs. 134 ± 35 mL/min/1.73 m^2^, *p* < 0.01) as compared to those whose follow-up time did not exceed 5 years at the study time (38 patients) (Fig. 4). Further analysis checked the relationships between risk groups (standard/intermediate and high), but no relationships were identified. We also found that the NGAL and NGAL/cr. ratio were positively correlated with BMI (*r* = 0.4, *p* < 0.01). No correlation was observed between high systolic and/or diastolic blood pressure and the investigated biomarkers.

Univariate analyses of KIM-1/cr. and NGAL/cr. with clinical parameters related to the applied treatment (cumulative doses of methotrexate and cyclophosphamide) did not show any significant differences. Summary of the analyzed parameters depending on the dosage of chemotherapeutic agents is presented in Table 4. Cut-off points were determined based on available studies and/or median and quartile ranges. Multivariate models assessing the relationship between KIM/cr. and NGAL/cr., potentially nephrotoxic factors such as cumulative dose of methotrexate, cyclophosphamide or abdominal radiotherapy, and reduced eGFR showed no differences.

**Table 4:** Biochemical parameters in acute lymphoblastic leukemia survivors according to the treatment used

	Methotrexate		*p*	Cyclophosphamide		*p*
Dose	< 10 g/m^2^	> 10 g/m^2^		< 4.5 g/m^2^	> 4.5 g/m^2^	
Number of patients	67	19		74	14	
	Mean ± SD	Mean ± SD		Mean ± SD	Mean ± SD	
Serum creatinine (mg/dL)	0.57 ± 0.18	0.59 ± 0.26	0.957	0.56 ± 0.20	0.59 ± 0.21	0.676
eGFR (mL/min/1.73m^2^)	121 ± 33	125 ± 33	0.780	122 ± 34	121 ± 29	0.851
Albuminuria (mg/L)	28.9 ± 152	16.6 ± 35	0.883	27.2 ± 146	17.5 ± 43	0.325
uKIM (ng/mL)	0.88 ± 0.8	0.71 ± 0.5	0.729	0.87 ± 0.8	0.67 ± 0.5	0.543
uKIM/cr. (ng/mg cr.)	7.02 ± 6.6	6.37 ± 3.6	0.787	7.04 ± 6.4	5.94 ± 3.8	0.717
uNGAL (ng/mL)	6.59 ± 6.4	5.98 ± 5.3	0.975	6.18 ± 6.2	8.01 ± 6.1	0.188
uNGAL/cr. (ng/mg)	59.6 ± 61.4	68.7 ± 76.6	0.715	57.1 ± 59.2	87.0 ± 88.5	0.114

## Discussion

The influence of anticancer treatment, especially radiotherapy and nephrotoxic agents such as ifosfamide and cisplatin, on kidney function is well known in patients with solid tumors treated in childhood [2, 3, 5]. This study is a consequence of our earlier reports suggesting that kidney abnormalities may occur in up to 25% of patients after leukemia treatment, in which none of the enrolled individuals developed serious signs of renal failure. In the current study we were trying to establish whether urinary KIM-1 and NGAL are elevated in survivors of childhood ALL patients compared to control group. Our previous research failed to show any changes in cystatin C level during and after the treatment for ALL; however, the sample size was small [24]. In the last years, new biomarkers indicating the development of AKI have been introduced. In adult patients, as well as in children treated for different cancers, elevated urine excretion of NGAL and KIM-1 was observed immediately or several days after the infusion of cisplatin or ifosfamide. Their concentrations increased with the severity of tubular injury [8, 18, 20]. Sterling et al. studied the changes in NGAL and Il-18 levels in the group of children treated with cisplatin, carboplatin and/or ifosfamide, and in the group with or without the symptoms of AKI [25]. They found higher NGAL levels after ifosfamide infusion in both AKI and non-AKI patients, but a greater and faster increase in biomarker levels was observed in AKI patients. This finding suggests that NGAL may be a useful predictor for detecting subclinical AKI. Similar studies, but only during intensive treatment, were carried out on the impact of methotrexate infusions. One of the first surveys was based on creatinine level during and immediately after MTX infusions. They indicated the detrimental effect of methotrexate on renal function by lowering creatinine clearance and increasing serum creatinine, especially in patients with delayed MTX elimination [26–28]. In a group of 20 children with newly diagnosed ALL, treated with MTX at a dose of 5 g/m^2^, Ylinen et al. observed an elevated cystatin C concentration 36 h after the infusion, but no changes in NGAL level were noted [29]. On the contrary, Li et al. in a group of children treated with different doses of methotrexate: 2, 3 and 5 g/m^2^ (according to the risk groups) found that patients who received doses higher then 3 g/m^2^ more often presented AKI with a parallel increase in urinary NGAL excretion. The authors suggested higher usefulness of the combination of serum creatinine ratio and serum NGAL (24 h after MTX infusion) for detecting AKI induced by a high dose of MTX compared with the serum creatinine ratio [11].

The results of NGAL and KIM-1 examinations in chronic kidney disease as markers of its development are controversial [12, 17, 30]. Seibert et al. in adult patients diagnosed for CKD in the course of the following diseases: diabetes mellitus, hypertensive nephropathy, glomerulonephritis and autosomal dominant polycystic kidney disease, did not confirm the better usefulness of NGAL and KIM-1as prognostic factors in comparison with standard albuminuria [12]. Other studies, on the contrary, suggest that these markers might be helpful as an indicator of AKI to CKD progression, corresponding to the histopathological changes in the kidneys or the degree of albuminuria in diabetic patients [14, 31]. In a study conducted in children with hyperuricemia, male, obese and hypertensive patients demonstrated higher urinary excretion of NGAL and KIM-1. The authors implied that this was probably a result of endothelial dysfunction, local inflammation in the kidneys or negative effect of hyperuricemia on renal tubules [32]. In turn, in children with diagnosed congenital hydronephrosis and normal kidney function, uKIM-1/cr. and uNGAL/cr. were elevated in comparison to healthy children, and after surgery the level of both markers gradually decreased [16]. Lobato et al. in children diagnosed with CKD, reported higher NGAL and KIM-1 levels in patients with progressive kidney insufficiency [33].

In cancer survivors treated during childhood for different neoplasms, a decline in kidney function was observed mainly after treatment with nephrotoxic cytostatics. Mulder et al. in a study carried out on a large cohort of CCS (1122 subjects), almost half of whom were patients treated in childhood for leukemia and lymphoma, found glomerular deterioration (GFR < 90 mL/min/1.73 m^2^) in 1.7% of patients after 15 years of diagnosis, and in 6.6% after 35 years [1]. They explained it as a consequence of physiological and pathological aging. They found no changes in GFR in survivors treated previously with high doses of MTX [1]. In another study by Mundi et al. in which the majority of participants were also patients after leukemia treatment, eGFR reduction, electrolyte disturbances, proteinuria, hematuria and/or hypertension after treatment with ifosfamide or nephrectomy were observed [34].

According to the Kidney Disease: Improving Global Outcomes (KDIGO) guidelines, CKD is defined by structural or functional abnormalities of the kidney for ≥ 3 months, with or without decreased eGFR or eGFR < 60 mL/min/1.73 m^2^ for ≥ 3 months, with or without kidney damage. An estimated GFR test may not correctly identify early symptoms or subtle progression of worsening kidney function. The assessment of serum creatinine levels has some limitations, including the influence of body lean mass, diet, patient’s physical activity and the time lag between the deterioration of kidney function and an increase in serum creatinine levels. Furthermore, some authors suggested that due to the curvilinear relationship between sCr and eGFR, the serum creatinine levels were increased when more than 40% of renal parenchyma was damage which may lead to the lack of detection of early stages of chronic kidney failure [35].

There is a need to discover new biomarkers, preferably non-invasive (especially in children) to predict deteriorating kidney function and identify patients in the early stages of the disease.

In our study, lowered eGFR values (< 90 mL/min/1.73 m^2^) were found in 13% of ALL survivors. We retrospectively checked data about eGFR measurement at least 3 months before the study time. All subjects with eGFR < 90 mL/min/1.73 during the study had also decreased eGFR three months earlier, but not < 60 mL/min/1.73 m^2^. None of participants met the criteria of CKD according to KDIGO guidelines. Considering the whole group of the patients studied, higher levels in urine NGAL (*p* < 0.05), NGAL/cr. ratio and KIM-1/cr. ratio were observed as compared to the control group (*p* < 0.0001). Higher values of NGAL/cr. (but not KIM-1/cr.) were found in the subgroup of patients with decreased eGFR in comparison with those who had normal eGFR; however, the sample size was small. ROC analyses revealed that among all analyzed parameters NGAL/cr. was the greatest predictor of decreased eGFR (< 90 mL/min/1.73 m^2^); however, the diagnostic profile was poor. It might be due to the low percentage of survivors with decreased eGFR and presumably quite young mean age at follow-up visits. We observed a correlation between NGAL and KIM-1 values but there was no correlation between the analyzed biomarkers and albuminuria. Lower eGFR and elevated tubular markers were found in ALL patients who survived more than 5 years after the end of treatment as compared to those whose follow-up time was less than 5 years. However, to determine whether the time after treatment affects the marker concentration, a longitudinal study should be performed on the same group of patients.

We did not observe the effect of age at diagnosis as well as the cumulative MTX dose. Higher levels of serum creatinine, KIM-1 and NGAL in urine and lower eGFR in the group with a longer follow-up (> 5 years) may predict worsening of kidney function many years after treatment. Elevated urine KIM-1 and NGAL levels indicate the possibility of subclinical tubular injury in those survivors who do not present “classical” chronic kidney disease as defined by KDIGO. These subtle changes in the eGFR, as well as in uNGAL and uKIM-1, may suggest the first subclinical sings of renal dysfunction and therefore may help identifying ALL survivors who are predisposed to progressive renal impairment. However, we realize that the time that has passed since the end of the therapy seems to be too short to draw final conclusions. Thus, further observational and longitudinal studies on the large cohort of ALL patients are required.

The trend towards higher NGAL level in women was observed in our study group and was confirmed by some authors in healthy children and adults [36]. Additionally, NGAL and NGAL/cr. levels correlated with BMI but no association was found with overweight and obese patients. This finding is in accordance with some results that elevated urinary excretion of NGAL may be associated with higher BMI [37–39].

In our opinion, this is the first study concerning the analysis of tubular injury biomarkers in acute lymphoblastic leukemia survivors. Previous studies on other markers provided divergent results. Grönroos et al. in a prospective, but small study (28 survivors), evaluated glomerular and tubular function. They found a gradual decrease in GFR during follow-up but no changes in tubular function, i.e., normal blood electrolyte levels and normal urine α1- and β-2-microglobulin. Median eGFR value after the end of follow-up was 113.9 mL/min/1.73m^2^, and only 11% of survivors had reduced GFR < 90 mL/min/1.73m^2^ [36]. Dekkers et al. did not observe glomerular and tubular dysfunction after MTX treatment in long-term survivors [41]. In one of the older studies, Bardi et al. noted normal cystatin C concentration and normal eGFR value, although elevated urinary NGAL was observed in 38% of leukemia/lymphoma survivors [42].

MTX is considered the main factor damaging kidney function; however, it has been suggested that this decline in renal function is reversible [5]. In our opinion, nephrotoxicity in childhood cancer survivors is multifactorial. Patients treated for leukemia are not only given MTX during anticancer treatment, but also, at the same time, they are exposed to other nephrotoxic drugs, such as aminoglycosides, vancomycin, amphotericin or contrast media, which all may aggravate kidney damage.

According to Grönroos et al. patients treated with methotrexate in combination with nephrotoxic antibiotics presented some late effects of deterioration of glomerular filtration and significant incidence of albuminuria [40]. Additionally, cancer treatment promotes dysfunction of different organs, metabolic processes inducing premature aging of cells and various organs, telomere shortening, production of proinflammatory cytokines and generates free-radicals, which cause direct damage to DNA. These factors are present in childhood cancer survivors and may affect the normal kidney function [43, 44].

There are several limitations of this study. It was a single-center analysis, with a relatively small number of patients. We did not evaluate the levels of urine NGAL and KIM-1 before the start of treatment, during exposure to chemotherapy or at the end of therapy, when possible recovery from MTX infusions may occur. The effects of other nephrotoxic drugs used in supportive care were not studied as well. The strengths of our research include the homogenous group of acute lymphoblastic leukemia survivors, relatively long follow-up time and no ethnic diversity.

In conclusion, the results of this study demonstrated that ALL survivors have higher levels of urine NGAL**,** NGAL/cr. and uKIM-1/cr. ratio compared to the control group. Further longitudinal, prospective analyses of NGAL and KIM-1 levels in ALL survivors and their potential role as monitoring tools for kidney dysfunction remain to be performed.
